# Fine‐scale prevalence and genetic diversity of urban small mammal‐borne pathogenic Leptospira in Africa: A spatiotemporal survey within Cotonou, Benin

**DOI:** 10.1111/zph.12953

**Published:** 2022-05-07

**Authors:** Henri‐Joël Dossou, Marine Le Guyader, Philippe Gauthier, Sylvestre Badou, Jonas Etougbetche, Gualbert Houemenou, Zouheira Djelouadji, Gauthier Dobigny

**Affiliations:** ^1^ Laboratoire de Recherche en Biologie Appliquée, Ecole Polytechnique d'Abomey‐Calavi Université d'Abomey‐Calavi Cotonou Benin; ^2^ Institut de Géographie, Aménagement du Territoire et Environnement Université d'Abomey‐Calavi Cotonou Benin; ^3^ Institut de Recherche pour le Développement Centre de Biologie pour la Gestion des Populations (UMR IRD, INRAE, Cirad, Institut d'Agronomie), MUSE Montferrier‐sur‐Lez France; ^4^ USC1233‐INRAE Rongeurs Sauvages, Risque Sanitaire et Gestion des Populations VetAgro Sup Marcy‐l'Etoile France

**Keywords:** health ecology, leptospirosis, rodents, urban ecology, zoonosis

## Abstract

Leptospirosis is a zoonotic disease that is caused by spirochete bacteria of the genus *Leptospira.* Around the world, one million people each year are infected, leading to 60,000 deaths. Infection occurs through contact with environmental pathogens excreted by mammals (notably rodents). Data on *Leptospira* and leptospirosis in Africa are rather scarce, especially in urban habitats though these appear to be favourable environments for the pathogen circulation and human contamination. Using qPCR, DNA sequencing as well as MST/VNTR approaches, we examined *Leptospira* occurrence and genetic diversity in 779 commensal small mammals that were sampled over 2 years in the city centre of Cotonou, Benin, from three neighbourhoods with contrasting socio‐environmental conditions. Overall prevalence reached 9.1%. However, very marked variations in both space and time were observed, with local peaks of high prevalence but no clear seasonal pattern. In most sites that could be regularly sampled, *Leptospira*‐positive rodents were found at least once, thus confirming the widespread circulation of the pathogen within small mammal communities of Cotonou. Interestingly, an unusual diversity of small mammal‐borne *Leptospira* species and genotypes was retrieved, with up to four species and three different genovars within the same neighbourhood, and even instances of two species and two genovars identified simultaneously within the same household. To our knowledge, such a high genetic diversity has never been described at such a fine scale, *a fortiori* in Africa and, more generally, within an urban environment. Altogether, our results underline that much remains unknown about leptospirosis as well as the associated infectious risk in African cities where the disease may be massively over‐looked.

**Impacts**

- Pathogenic *Leptospira* were monitored in small mammals from three socio‐environmentally contrasted neighbourhoods of Cotonou city centre, Benin, during a 2 year‐long survey.
- Small mammal‐borne pathogenic *Leptospira* prevalence varies greatly in both space and time within Cotonou city, Benin, with some high local peaks but no obvious seasonal patterns.
- Unusually elevated *Leptospira* species and genovar diversities were observed at the city‐, neighbourhood‐ and even household‐scales, thus representing a rare example of *Leptospira* species and genovar coexistence at a very fine scale.

## 1 INTRODUCTION

Leptospirosis infects one million and kills 60,000 people each year (Costa et al., 2015). The etiologic agents, spirochetes bacteria of the genus *Leptospira*, multiply in renal tubules of animal reservoirs, especially rodents, and are subsequently excreted in the environment (reviewed in Haake & Levett, 2015). *Leptospira* gathers nine currently recognized human‐pathogenic species and 350 serologically distinguishable serovars (review in Karpagam & Ganesh, 2020). Animals and humans become infected following contact with contaminated soils and waters Thus, breeding and water‐related activities and events (e.g. rice and market garden production, water‐based recreational activities, flooding episodes) are important risk factors for infection (Mwachui et al., 2015; review in Karpagam & Ganesh, 2020). Poor and environmentally degraded urban areas such as slums have also been associated with high leptospiral prevalence, with the case of Pau da Lima favela in Salvador, Brazil, being particularly well documented (e.g. Ko et al., 1999; Reis et al., 2008; Cassanovas‐Massana et al., 2018; reviewed in Cornwall, 2016). However, in this latter case, the infectious system is relatively simple, with one single rodent reservoir, *Rattus norvegicus*, shedding the *Leptospira interrogans* serovar Copenhageni that is responsible for most human leptospirosis cases (Kô et al., 1999; Tucundava da Faria et al., 2008; Costa et al., 2014). In Rio de Janeiro, Brazil, brown rats are also considered the key reservoir of the urban cycle of *L. interrogans* serovar Icterohaemorrhagiae that is dominant in the city (Martins & Lilenbaum, 2013). In the same manner, only one clonal genotype was found in rats from Johannesburg, South Africa (*L. borgpetersenii* Javanica; Moseley et al., 2020). However, in other settings, several *Leptospira* species and serovars may coexist, thus making the ecology of the disease probably much more complex, and the investigation of *Leptospira* species and serovars crucial (Guernier et al., 2017). For instance, in the city of Lyon, France, one single species (*L. interrogans*) but two serovars (Icterohaemorragiae and Copenhageni) were found in *Rattus norvegicus* from eight different sites of the city (Ayral et al., 2015). In Malaysian towns, three species of *Rattus* (*R. rattus*, *R. norvegicus* and *R. exulans*) were found to harbour two *Leptospira* phylogenetic lineages and two serovars (*L. borgpetersenii* serovar Javanica and *L. interrogans* serovar Bataviae) (Benacer et al., 2016). In the same manner, *L. interrogans* and *L. kirschneri* were identified in only three house mice, brown and black rats in Kibera slum, Nairobi, Kenya (Halliday et al., 2013). The dynamics of leptospires circulation in the environment may even rendered even more complex by co‐infection events: in Madagascar, several cases of co‐infections by up to three different *Leptospira* species (*L. interrogans*, *L. borgpetersenii* and *L. mayottensis*) were observed in various animal reservoirs, with a probable spill‐over of *L. mayottensis* from endemic rodents to the invasive black rat (Moseley et al., 2018).

The coastal region of West Africa along the Guinea Gulf is experiencing an accelerated urbanization (i.e. the so‐called Abidjan‐Lagos corridor; Choplin, 2019), where hundreds of thousands of urban dwellers live in socio‐environmentally degraded conditions. In this region, leptospiral burden, though under‐documented, is expected to be important (Dobigny et al., 2018). Contrary to what is usually observed in European and American towns where only rats and mice coexist, the small mammal communities from the coastal West African city centres still harbour native species due to the ongoing but incomplete bio‐invasion of cosmopolitan rodents (e.g. Garba et al., 2014). As such, south Benin cities are characterized by (i) the co‐occurrence of invasive black and Norway rats with the native *Mastomys natalensis* as well as, to a lesser extent, *Cricetomys gambianus* and *Praomys derooi*; and (ii) the significative prevalence of the native shrew *Crocidura olivieri* in small mammal assemblages (>20% of all captures in Cotonou) (Hima et al., 2019; Houéménou et al., 2019). We recently provided an overview of rodent‐ and shrew‐borne leptospires diversity in this part of the country (Houéménou et al., 2019). It was demonstrated that several *Leptospira* species (*L. interrogans*, *L. kirschneri* and *L. borgpetersenii*) were circulating in both the native and invasive mammalian hosts. It was also suggested that some kind of host‐specificity may exist and that local spatiotemporal variations in prevalence was important. In the current study, we build on these results by conducting a long term study, sampling at a finer scale: we sampled in three neighbourhoods of Cotonou, investigating small mammal‐borne leptospires diversity and ecology at both the species and serovar levels during a period of 2 years. To our knowledge, the present study is the first one to provide data on *Leptospira* genotypes in Benin.

## 2 MATERIAL AND METHODS

### 2.1 Field experimental design and small mammal trapping

**FIGURE 1:**
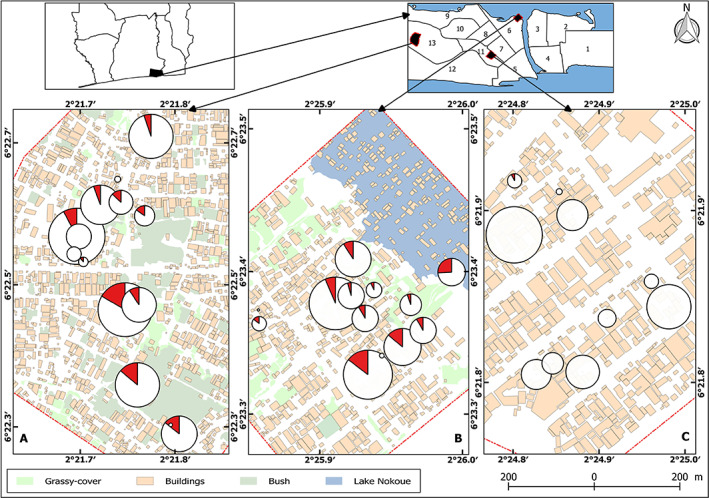
Spatial distribution of the small‐mammal‐borne pathogenic *Leptospira* prevalence in Agla panel a on the left), Ladji (panel b in the middle) and saint‐Jean (panel c on the right) neighbourhoods. Circle sizes are proportional to the number of investigated animals, while red pie charts represent the frequencies of qPCR‐positive ones. Data were pooled across temporal sessions. Landscapes were reconstructed following in‐site android‐based mapping based on *KoBoCollect* (i.e. APK v.1.23.3) and *open street map* (i.e. ODK collect v.1.7.1 and OSM tracker v.0.6.11; see Dossou et al., submitted, for further details) toolbox suite as well as GIS‐processing under QGis v.2.18.14

Three districts of Cotonou, Benin, were selected according to their contrasting socio‐environmental profiles (Dansou, 2006; Sotindjo, 2009; PPCU3C, 2010): Agla, Ladji and Saint Jean (Figure 1). Agla is a relatively recent district with poorly sanitation (NB: it currently benefits from various urban management actions that were started after the present study). Extensive parts of this wide shallows get flooded at the beginning of the great rainy season (May–July) due to rainfall accumulation. Ladji is a very poor, densely populated and insalubrious neighbourhood, which is located along the edge of Lake Nokoué. This area gets partly flooded during the second half of the great rainy season (June and July) until the end of the short rainy season (September–October) following an increase of the lake level. Saint‐Jean is a formal district of colonial origin with a mix of hard‐built and precarious habitats. It is not prone to flooding per se, but rainfall may create large ephemeral ponds.

Explicit oral agreements were systematically obtained with local traditional (e.g. family and household heads, shop, firm and garden owners) as well as administrative (i.e. Cotonou City Hall services, urban district chiefs) authorities before rodent trapping. None of the rodent species captured for this study has protected status according to IUCN/CITES. All animals were treated in a human manner in accordance with the guidelines of the American Society of Mammalogists (Sikes & Gannon, 2011). Field work in Benin was conducted under a research agreement signed between Republic of Benin and the French Institute of Research for Development (30th, September 2010). According to the Nagoya protocol, access and sharing of benefits was agreed by the government of the Republic of Benin (file 608/DGEFC/DCPRN/PF‐APA/SA). Note that no ethical committee agreement is required in Benin to conduct research on pest animals such as those described in the present study.

Rodent captures were conducted concomitantly (i.e. within the same 3–5 weeks) in Agla (1,728 trap nights in total; 1,155 indoor and 573 outdoor), Ladji (1,744 trap nights; 1,603 indoor and 141 outdoor) and Saint‐Jean (1,791 trap nights; 1,184 indoor and 607 outdoor) during six successive periods: in November–December 2016, March 2017, June 2017, October 2017, February–March 2018 and June 2018, thus encompassing the beginning (June) and end (October–December) of the rainy season as well as the dry season (February and March). In each district, the same households (10–12) were sampled during each period, both inside (i.e. bedrooms, kitchens, store rooms, etc.) and outside (i.e. courtyards). Locally made wire‐mesh traps were used during the first session (November–December 2016) while both Sherman (8 × 9 × 23 cm; ©Sherman) and locally made wire‐mesh traps (10 × 10 × 25 cm) were used together for all subsequent sessions (March 2017 to June 2018). The use of these two types of traps was important since different species may be preferentially captured depending on the trap type (Garba et al., 2014). Baits were made of a mixture of peanut butter and fish. Traps were set for three consecutive nights and checked each morning. When a trap captured an individual, it was replaced by a new one, while empty traps were rebaited.

Rodents were brought alive to the laboratory where they were always processed on the day of capture. They were euthanized using diethyl‐ether and cervical dislocation. General morphology and external measurements were used to provide a preliminary taxonomic identification. Many African rodent genera can sometimes be difficult to distinguish on morphological grounds alone (Granjon & Duplantier, 2009). For this reason, a particular attention was paid to species‐specific identification. To this end, all *Rattus* individuals were also identified using a genotyping method as well as cytochrome b DNA sequencing (Dobigny et al., 2008 and Berthier et al., 2016). In addition, all *Praomys* individuals, at least one *Mastomys* individual per site per session, the single house mouse captured (*Mus musculus*), as well as one giant African rat (*Cricetomys*) and one shrew (*Crocidura*) per district were unambiguously identified through cytochrome b gene sequencing from kidney‐ or spleen‐extracted DNA (data are not shown, but see Dobigny et al., 2008, and Berthier et al., 2016, for DNA sequencing and genotyping procedures). Reference datasets for barcoding‐based taxonomic identifications were built from hundreds of precisely documented West African rodent specimens housed in the Centre of Biology for Population Management (CBGP) small mammal collections (see Dobigny et al., 2008) as well as from published data (e.g. Olayemi et al., 2012 for *Cricetomys*; Jacquet et al., 2015 for *Crocidura*; Mikula et al., 2020 for *Praomys*).

One kidney and spleen fragments were collected on all animals, 96° ethanol‐preserved andall deposited in the CBGP sample collections, Montpellier, France (https://doi.org/10.15454/WWNUPO), with duplicated 96° ethanol‐preserved spleens also deposited at the URIB, Abomey‐Calavi University, Cotonou, Benin.

### 2.2 DNA extraction and qPCR‐based detection of pathogenic *Leptospira*

Genomic DNA was extracted from one fragment of ethanol‐preserved kidney tissue samples using 96‐Well Plate Animal Genomic DNA Mini‐Preps Kit (Biobasics^©^) according to the manufacturer's recommendations. DNA was eluted using 150 μl elution buffer. Detection of pathogenic *Leptospira* was performed following a probe‐based qPCR approach that targets a fragment of the *LipL32* gene, using a LightCycler® 480 (Roche Diagnostics, France) and according to previously described protocols (Dobigny et al., 2015). All experiments were conducted in duplicate in 384‐well micro‐titre plates with 2 μl DNA in a 7 μl final volume for each reaction. On each qPCR plate, a standard curve was made using *Leptospira interrogans* serovar Icterohaemorragiae strain Verdun DNA extracted from culture (Spirochete Laboratory, Pasteur Institute of Paris, National Reference Center for Leptospirosis and WHO Collaborative Centre). Extraction (no animal tissue) and qPCR reaction (i.e. molecular biology grade water instead of DNA) negative controls were systematically added to each plate.

### 2.3 *Leptospira* molecular characterization

*Leptospira* species identification relied on 16S rDNA barcoding. To do so, amplification was performed following published protocols with slight modification (Mérien et al., 1992): conventional PCRs were performed in a final volume of 50 μl containing 35 μl of H_2_O, 5 μl of 10× buffer (Qiagen, Courtaboeuf, France), 2 μl of 25 mM MgCl_2_, 1 μl of 10 mM dNTPs (Qiagen), 1 μl of forward primer (10 μM), 1 μl of reverse primer (10 μM), 0.5 μl of HotStarTaq DNA Polymerase (Qiagen) and 5 μl of target DNA. The following program was used: a 15‐min enzyme activation step at 95°C, followed by 40 cycles of 95°C for 30 s, 57°C for 30 s and 72°C for 1 min with a final elongation step of 72°C for 10 min. PCR mix without the target DNA and PCR mix with *Leptospira interrogans* serovar Copenhageni strain Fiocruz DNA were included as negative and positive controls, respectively. Amplified products were sent for sequencing (@Genoscreen) and *Leptospira* sequences were aligned using genomic tools freely available on the PRABI‐Gerland bioinformatic platform website (https://npsa‐prabi.ibcp.fr/cgi‐bin/npsa_automat.pl?page=/NPSA/npsa_multalinan.html), and identified using Nucleotide BLAST search implemented in NCBI (http://blast.ncbi.nlm.nih.gov) as well as genetic similarity with reference *Leptospira* DNA sequences available in the VetAgro USC1223 Lab (see Merien et al., 1992).

Molecular typing was performed using two complementary approaches, namely the multi‐locus Variable Number Tandem Repeat (VNTR) and Multi‐spacer Sequence Typing (MST) methods. First, VNTR analysis was conducted on loci VNTR4, VNTR7, VNTR10 and VNTR Lb4 as described in Salaün et al. (2006). In brief, 5–7 μl of DNA were added to 45 μl of PCR mix (^©^Qiagen) for PCR amplifications with 40–45 cycles. Annealing temperatures were 52°C for VNTR4 and VNTR10, 54°C for VNTR7 and 53°C for VNTRLb4. Second, samples identified as belonging to the *L. interrogans* serogroup Icterohaemorrhagiae by the VNTR analysis were further characterized using the discriminating MST3 locus. We relied on the protocol previously described by Zilber et al. (2014) with an annealing temperature of 50°C. Amplification products were sent for sequencing (^©^Genoscreen), and the resulting sequences were compared to the MST reference database (Zilber et al., 2014). VNTR and MST3 profiles were used in combination to deduce *Leptospira* genotype and putative serovar assignation.

Pearson's *χ* ^2^ tests were performed to compare prevalence between host species, districts and trapping sessions. Seasonal prevalence was also compared using a Pearson's *χ* ^2^ test, with February/March, June and October/November sessions of years 1 and 2 being pooled, but they were further investigated through a Kruskal‐Wallis comparison of the prevalence measured in Agla and Ladji households between seasons (with years 1 and 2 pooled). All statistics were conducted under R v.3.5.0 (R consortium, 2018).

## 3 RESULTS

### 3.1 Small mammal diversity and distribution

In total, 779 small mammals were collected in Agla, Ladji and Saint‐Jean during six campaigns that took place between November 2016 and June 2018. Among them, *Rattus rattus* was the most dominant species (*N* = 456), followed by the shrew *Crocidura olivieri* (*N* = 164), then *Mastomys natalensis* (*N* = 88), *Rattus norvegicus* (*N* = 42), *Cricetomys gambianus* (*N* = 17), *Praomys derooi* (*N* = 11) and one single house mouse (*Mus musculus*). *Rattus rattus* was found to be abundant and widespread in all three districts, with black rats trapped at least once in 31 out of 35 (88.6%) of the prospected households (Table 1 and Table S1). In the same manner, shrews were quite numerous and present at least once in 29 (82.9%) houses from all three districts. The native commensal *Mastomys natalensis* was mainly captured in Agla (70.5% of all *Mastomys natalensis* captures) with most individuals (79%) captured in two neighbouring households (see sites S‐AGL‐5 and S‐AGL‐5′ in Table S1). Due to the inadequate size of the traps, giant pouched rats were found on only twelve occasions, eleven of which were in Saint Jean. *Praomys derooi* were rare (*N* = 11) and trapped only in Saint Jean. Finally, only a single house mouse was captured in Ladji. Sample sizes were quite similar among temporal sessions, ranging from 119 (March 2017) to 146 (June 2018). In the same manner, total numbers of animals captured within each district were similarly high (*N* = 224, *N* = 249 and *N* = 306 in Saint Jean, Ladji and Agla, respectively; Table 1). The trapping results detailed by district, session and species are provided in Table 1. Details by trapping sites (i.e. households) are provided in the Table S1.

**TABLE 1:** Captures and prevalence by session, neighbourhood and species

	All		Rra		Rno		Mna		Pde		Cga		Mmu		Cro	
	*N*	pos (%)	*N*	pos (%)	*N*	pos (%)	*N*	pos (%)	*N*	pos (%)	*N*	pos (%)	*N*	pos (%)	*N*	pos (%)
Nov–Dec 2016																
Agla	51	9 (17.6)	37	8 (21.7)	3	1 (33.3)	10	0 (0)	0	–	0	–	0	–	1	0 (0)
Ladji	36	4 (11.1)	31	3 (9.7)	1	1 (100)	1	0 (0)	0	–	0	–	0	–	3	0 (0)
Saint Jean	36	0 (0)	32	0 (0)	0	–	1	0 (0)	0	–	2	0 (0)	0	–	1	0 (0)
All 3 districts	123	13 (10.6)	100	11 (11)	4	4 (50)	12	0 (0)	0	–	2	0 (0)	0	–	5	0 (0)
March 2017																
Agla	47	2 (4.3)	29	2 (6.9)	1	0 (0)	13	0 (0)	0	–	0	–	0	–	4	0 (0)
Ladji	36	0 (0)	34	0 (0)	1	0 (0)	0	–	0	–	0	–	1	0 (0)	0	–
Saint Jean	36	0 (0)	22	0 (0)	0	–	9	0 (0)	2	0 (0)	0	–	0	–	3	0 (0)
All 3 districts	119	2 (1.7)	85	2 (2.4)	2	0 (0)	22	0 (0)	2	0 (0)	0	–	1	0 (0)	7	0 (0)
June 2017																
Agla	59	8 (13.6)	20	1 (5)	10	3 (30)	19	4 (21.1)	0	–	1	0 (0)	0	–	9	0 (0)
Ladji	38	2 (5.3)	27	1 (3.7)	2	0 (0)	1	1 (100)	0	–	0	–	0	–	8	0 (0)
Saint Jean	30	0 (0)	19	0 (0)	0	–	2	0 (0)	0	–	2	0 (0)	0	–	7	0 (0)
All 3 districts	127	10 (7.9%)	66	2 (3)	12	3 (25)	22	5 (22.8)	0	–	3	0 (0)	0	–	24	0 (0)
Oct 2017																
Agla	44	5 (11.4)	27	0 (0)	5	3 (60)	5	1 (20)	0	–	0	–	0	–	7	1 (14.3)
Ladji Saint	43	1 (2.3)	29	1 (3.4)	2	0 (0)	3	0 (0)	0	–	0	–	0	–	9	0 (0)
Jean	38	0 (0)	25	0 (0)	0	–	5	0 (0)	1	0 (0)	5	0 (0)	0	–	2	0 (0)
All 3 districts	125	6 (4.8)	81	1 (1.2)	7	3 (42.9)	13	1 (7.7)	1	0 (0)	5	0 (0)	0	–	18	1 (5.6)
Feb–March 2018																
Agla	47	9 (19.1%)	24	5 (20.8)	2	1 (50)	6	0 (0)	0	–	0	–	0	–	15	3 (20)
Ladji	46	23 (50)	28	12 (42.9)	7	7 (100)	0	0 (0)	0	–	0	–	0	–	11	4 (36.4)
Saint Jean	46	1 (2.2)	17	0 (0)	0	–	2	0 (0)	4	0 (0)	5	1 (20)	0	–	18	0 (0)
All 3 districts	139	33 (23.7)	69	17 (24.6)	9	8 (88.9)	8	0 (0)	4	0 (0)	5	1 (20)	0	–	44	7 (15.9)
June 2018																
Agla	58	5 (8.6)	18	3 (16.7)	6	2 (33.3)	9	0 (0)	0	–	0	–	0	–	25	0 (0)
Ladji	50	5 (10)	28	2 (7.1)	2	1 (50)	0	–	0	–	0	–	0	–	20	2 (10)
Saint Jean	38	0 (0)	9	0 (0)	0	–	2	0 (0)	4	0 (0)	2	0 (0)	0	–	21	0 (0)
All 3 districts	146	10 (6.8)	55	5 (9.1)	8	3 (37.5)	11	0 (0)	4	0 (0)	2	0 (0)	0	–	66	2 (3)
Total																
Agla	306	35 (11.4)	155	19 (12.3)	27	10 (37)	62	5 (8.1)	0	–	1	0 (0)	0	–	61	4 (6.6)
Ladji	249	35 (14.1)	177	19 (10.7)	15	9 (60)	5	1 (20)	0	–	0	–	1	0 (0)	51	6 (11.8)
Saint Jean	224	1 (0.4)	124	0 (0)	0	–	21	0 (0)	11	0 (0)	16	1 (6.3)	0	–	52	0 (0)
All 3 districts	779	71 (9.1)	456	38 (8.3)	42	19 (45.2)	88	6 (6.8)	11	0 (0)	17	1 (5.9)	1	0 (0)	164	10 (6.1)

Note

‘Rra’, ‘Rno’, ‘Mna’, ‘Pde’, ‘Cga’, ‘Mmu’, ‘Cro’ and ‘pos’ stand for *Rattus rattus*, *R. norvegicus*, *Mastomys natalensis*, *Praomys derooi*, *Cricetomys gambianus*, *Mus musculus*, *Crocidura olivieri* and qPCR‐positive individuals, respectively.

### 3.2 *Leptospira* prevalence in small mammals

Overall pathogenic *Leptospira* prevalence in small mammal was 9.1% (74/779). In 13 cases (out of 74), only one Ct value (for ‘Cycle threshold’, sometimes referred to as Cp, the ‘Crossing point’; that is the number of amplification cycles above which fluorescence exceeds background signal) could be obtained, while Ct values could be calculated for both duplicates for the remaining 61 *Leptospira*‐positive individuals. Ct values ranged between 20.72 and 38.16, with an average Ct value of paired duplicates of 31.05. Species‐specific average Ct values (including both duplicates) were 28.29, 28.31, 29.56, 34.3 and 32.31 in *Mastomys natalensis*, *Rattus norvegicus*, *Cricetomys gambianus* (one single positive individual, hence two replicates and two C_*t*_ values), *Crocidura olivieri* and *Rattus rattus*, respectively. The standard deviation between pairs of duplicate C_*t*_ values ranged between 0.01 and 1.98, with an average of 0.46, thus showing a good convergence between qPCR duplicates.

Important variations were observed among host species as well as in both space and time (Table 1 and Table S1). Significant differences in Leptospira prevalence were observed depending on the host species (Pearson's *χ* ^2^ test, *p* < 4.05e‐12): the host species with the highest prevalence was *Rattus norvegicus* with 19 individuals out of 42 (45.2%) carrying pathogenic leptospires. The other species showed much lower prevalence: 8.3% (38/456) in *Rattus rattus*, 6.9% (6/88) in *Mastomys nalalensis*, 6.1% in *Crocidura olivieri* (10/164) and 5.9% (1/17) in *Cricetomys gambianus*. No *Leptospira* DNA was detected in the two least sampled species, namely *Praomys derooi* (*N* = 11) and *Mus musculus* (*N* = 1).

**FIGURE 2:**
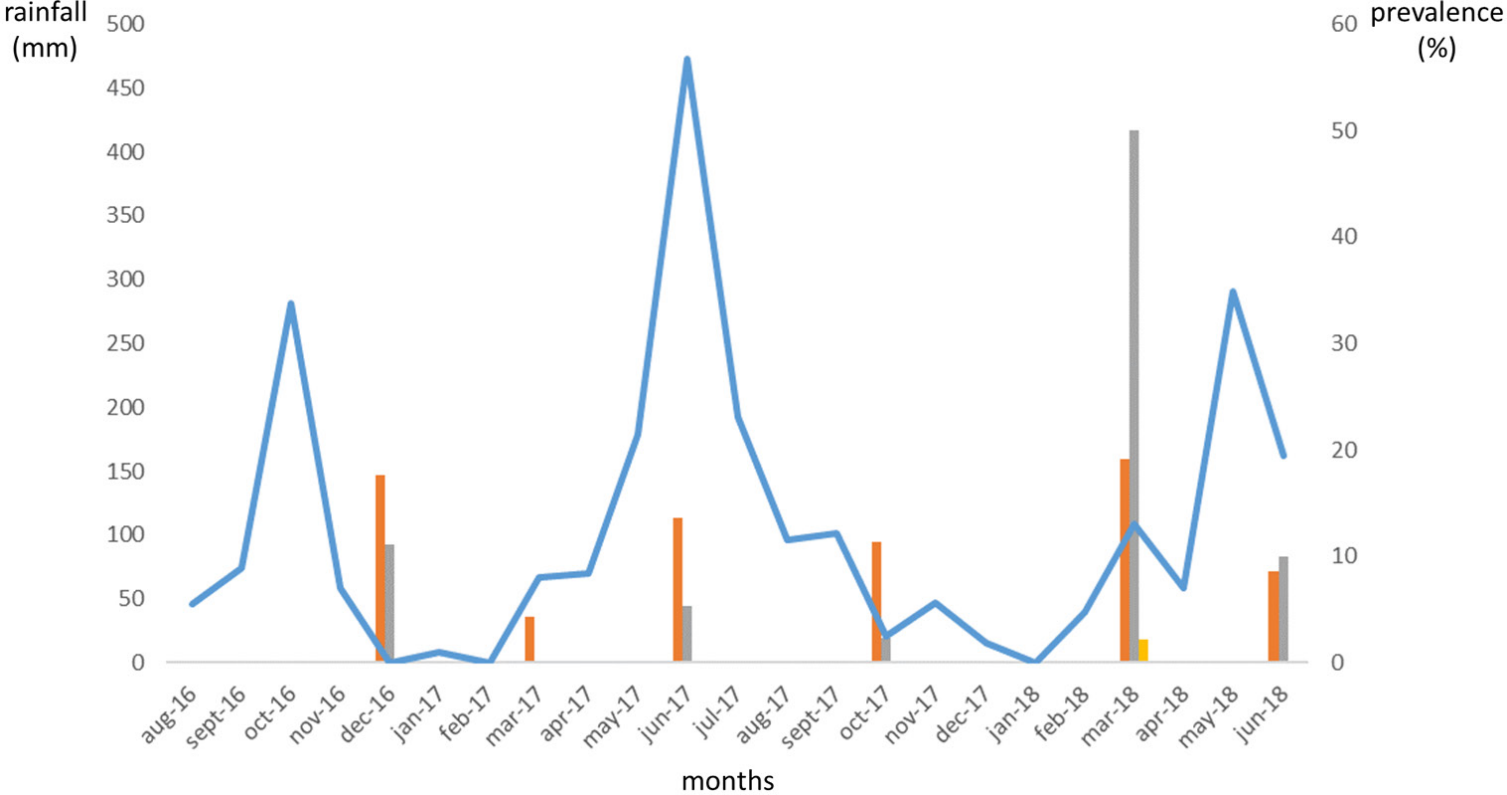
Small‐mammal‐borne pathogenic *Leptospira* prevalence (in %, right *y*‐axis) and monthly rainfall (in millimetres, left *y*‐axis) from august 2016 to June 2018 (*x*‐axis). Prevalence in Agla, Ladji and saint‐Jean are presented as histograms in orange, grey and yellow colour, respectively. The blue line shows monthly rainfall variations across the study period

Temporal variations were very marked, too, ranging from 1.7% of cumulative prevalence in March 2017 (*N* = 199) to 23.7% in February–March 2018 (*N* = 139) (Figure 2). These differences were highly significant between sessions (Pearson's *χ* ^2^ = 46.2, *df* = 5, *p* = 8.21e9). However, when considering Agla and Ladji, no significant seasonal pattern could be observed (Kruskal‐Wallis tests, *p* > 0.32) with total minimal and maximal prevalence being observed at the same period in two different years (March 2017 and February–March 2018) (Figure 2). This was also true when considering only one given species (e.g. *Rattus rattus* and *R. norvegicus*) or one given district (i.e. Agla and Ladji). Atypically high prevalence was observed in February–March 2018 during which the maximal values of the whole survey were detected for Agla, Ladji, Saint‐Jean among *Rattus rattus*, *R. norvegicus*, *Cricetomys gambianus* and *Crocidura olivieri* species. Agla showed higher prevalence than Ladji during the four first sessions (from October–November 2016 to October 2017), while the reverse was true for the two last ones (March and June 2018) (Figure 2). The maximal prevalence was retrieved in February–March 2018 in Ladji, where 23 out of the 46 small mammals investigated (50%) were found *Leptospira*‐positive (Figure 2). Among these were: 12 out of 28 (42.9%) black rats, all seven Norway rats (100%) as well as 4 out of 11 (36.4%) shrews.

Spatial differences were very strong with 11.4% and 14.1% of cumulative prevalence observed in Agla and Ladji, respectively, while only one single small mammal (*Cricetomys gambianus*, in February–March 2018) was found positive in Saint Jean (0.4%) (Figure 2; Pearson's *χ* ^2^ test, *p* = 3.66e‐8). Differences in the two most prevalent areas, namely Agla and Ladji, were not significant (*χ* ^2^ test, *p* = .14). Significant spatial variations were also observed at a very local scale (Pearson's *χ* ^2^ test, *p* < .009), with 2‐year long site‐specific prevalence ranging from 0% (4 sites) to 53% (0%–25% in Agla, and 0%–53% in Ladji; Figure 1; see also Table S1 and Figures [Link], [Link], [Link]). During the whole survey, *Leptospira*‐positive small mammals were found at least once in 21 out of 37 (56.8%) households; this value increases to 21 out of 27 (77.8%) when one considers only the two most insalubrious and flood‐prone districts Agla (10 out of 14, 71.4%) and Ladji (11 out of 13, 84.6%).

### 3.3 *Leptospira* species and genotypes

A total of 70 *Leptospira*‐positive samples (see Table 2) could be successfully assigned to *Leptospira* species through 16S rDNA sequencing and 100% genetic similarity with GenBank hits (reference sequences available in GenBank under accession numbers OM283612‐OM283615)*. L. interrogans* was the most represented species (43/70, 61.4%), followed by *L. borgpetersenii* (24/70, 34.3%), *L. kirschneri* (1/70, 1.4%) and *L. broomi* (1/70, 1.4%). In one sample, a mixture of *L. interrogans* and *L. borgpetersenii* was observed (1.4%).

**TABLE 2:** Distribution of *Leptospira* species and serovars across reservoir species (a, b), neighbourhoods (a, b) and session (c)

a				
	*Leptospira* species			
	Agla	Ladji	St Jean	TOT
Rra	10 bor + 9 int + 1 broo	4 bor + 14 int + 1 kir	–	14 bor + 23 int + 1 broo +1 kir
Rno	2 bor + 6 int	3 bor + 6 int	–	5 bor + 12 int
Cro	1 bor + 2 int	4 int	–	1 bor + 6 int
Mna	4 bor + 1 int/bor	1 int	–	4 bor + 1 int + 1 int/bor
Cri	–	–	1 int	1 int

b				
	*Leptospira* serovars			
	Agla	Ladji	St Jean	TOT
Rra	4 copA + 6 tara/java	1 copA + 1 copB + 7 ict	–	5 copA + 6 tara/java + 7 ict + 1 copB
Rno	5 copA	2 copB + 2 tara/java + 4 ict	–	5 copA + 2 copB + 2 tara/java + 4 ict
Cro	–	1 ict	–	1 ict
Mna	–	1 ict	–	1 ict
Cri	–	–	1 copB	1 copB

c						
	*Leptospira* species and serovars across sessions					
	Total	Interrogans	Borgpetersenii	Kirschneri	Broomi	Int + borg
Nov‐16	13 (8 sérovars)	6 (4 copA + 2 copB)	7 (2 tara)			
March‐17	2 (2 sérovars)	1 (1 copA)	1 (1 tara)			
June‐17	10 (3 sérovars)	4 (2 copA + 1 ict)	5		1	
Oct‐17	6 (2 sérovars)	1 (1 copA)	2 (1 tara)	1		1
Feb‐18	32 (16 sérovars)	29 (2 copB + 12 ict)	3 (2 tara)			
June‐18	8 (4 sérovars)	2 (2 copA)	6 (2 tara)			

Note

‘Bor’, ‘int’, kir’ and ‘broo’ stand for *Leptospira borgpeterseni*, *L. interrogans*, *L. kirschneri* and *L. broomi*, respectively. ‘copA’, ‘copB’, ‘ict’ and ‘tara/java’ correspond to serovars copenhageni a, copenhageni B, icterohaemorragiae and tarassovi/javanica, respectively.

All small mammal species were found infected by sereval *Leptospira* species: four species were identified in the most represented reservoir, *Rattus rattus* (*L. interrogans*, *L. borgpetersenii*, *L. kirschneri* and *L. broomi*). Two species (*L. interrogans* and *L. borgpetersenii*) were detected on *R. norvegicus*, *Mastomys natalensis* and *Crocidura olivieri*. The only qPCR‐positive *Cricetomys gambianus* was infected by *L. interrogans*. The distribution of *Leptospira* species per host taxon and district is detailed in Table 2. At a more local level, two different *Leptospira* species were found in 6/10 and 3/10 households of Agla and Ladji, respectively (data are not shown). The coexistence of mammal hosts carrying different *Leptospira* species within the same household was found twice during the same trapping session: among four positive black rats caught in November 2016 within the S‐AGL‐9 site in Agla, three were carrying *L. interrogans* and one was carrying *L. borgpetersenii* (data are not shown). In the same manner, in site S‐LAD‐8 investigated on February 2018, six positive small mammals were infected by *L. interrogans* (two Norway rats and two shrews) and *L. borgpetersenii* (two Norway rats) (data are not shown).

Among the 70 qPCR‐positive samples that could be sequenced, 35 could be further investigated for genotyping (Table 2). In total, four genotypes could be identified by the combination of VNTR and MST analyses: 3 profiles belong to the Icterohaemorrhagiae genogroup, and one to the Tarassovi or Javanica genogroup (which display the same VNTR profile). These profiles could be assigned to *L. interrogans* genovars Icterohaemorragiae (13/35, 37.1%), *Copenhageni* genotype Shibaura 9 (10/35, 28.6%) and Copenhageni genotype M20 (4/35, 11.4%) on the one hand, and to the *L. borgpetersenii* profile that resembles genovars Tarassovi kanana, Javanica ceylonica or Javanica dehong—which could not be unambiguously distinguished from each other (Table 2b,c). All four genotypes were found at least once in the two *Rattus* species. Three of them were identified in only one single representatives of *Mastomys natalensis* (*L. interrogans* Icterohaemorragiae), *Crocidura olivieri* (*L. interrogans* Icterohemorragiae) and *Cricetomys gambianus* (*L. interrogans* Copenhageni M20) (Table 2b).

Coexistence of genotypes within the same household during the same trapping period mirrors the pattern described above for *Leptospira* species, namely three black rats carrying *L. interrogans* Copenhageni Shibaura 9 and one black rat carrying *L. borgpetersenii* Tarassovi/Javanica in site S‐AGL‐9 in November 2016 on the one hand; two Norway rats and one shrew carrying *L. interrogans* Icterohemorragiae and one Norway rat carrying *L. borgpetersenii* Tarassovi/Javanica in site S‐LAD‐8 in February 2018 on the other hand (data are not shown). Interestingly, in spite of quite similar sample sizes, two serovars were shared between Agla and Ladji (9 and 1 *Copenhageni*, respectively; 6 and 2 Tarassovi/Javanica, respectively) while one was exclusively found in Ladji (Icterohaemorragiae). Genovar Copenhageni M20 was found in Ladji (*N* = 3) and Saint Jean (one single positive rodent), but was absent from the Agla sample (Table 2b).

Genovars from the *L. kirschneri* and *L. broomi* samples could not be identified since the genetic profile was incomplete for the former, and there are no reference profiles available for *L. broomi*.

Relative abundances of the most prevalent *Leptospira* species, namely *L. interrogans* and *L. borgpetersenii*, according to temporal sampling sessions, were quite similar for the four first campaigns, while *L. interrogans* was dominant in February 2018 (29 *L. interrogans* vs. 3 *L. borgpetersenii*) and *L. borgpetersenii* was more common in June 2018 (2 *L. interrogans* vs. 6 *L. borgpetersenii*). However, sample sizes were quite low, thus precluding robust analyses and interpretation. No seasonal trend was observed at the genovar/genotype level, with *L. interrogans* genovars Copenhageni Shibaura 9, Copenhageni M20 and Icterohemorragiae being observed at five, two and two temporal sessions, respectively, and during both rainy and dry seasons (Table 2c). Once again, however, no quantitative analysis was conducted on genovar/genotype prevalence variation in time due to loo low sample size.

## 4 DISCUSSION

We provide new African data on rodent‐borne *Leptospira*, which are lacking for many countries on the continent (De Vries et al., 2014; Allan et al., 2015), especially in urban settings where only very limited investigations have been conducted (e.g. Durban, Cape Town and Johannesburg, South Africa: Taylor et al., 2008, Moseley et al., 2020; Malagasy cities: Rahelinirina et al., 2010; Nairobi, Kenya: Halliday et al., 2013; Niamey, Niger: Dobigny et al., 2015). In Benin, it has already been shown that mean prevalence of rodent‐borne leptospires along the extensively urbanizing coastal corridor was 12.9% (Houéménou et al., 2019), with an overall prevalence in the city of Cotonou between 12% and 13% (Houéménou et al., 2013, 2019). However, important variations in both space and time were noted, suggesting that local conditions were critical to explain *Leptospira* circulation in domestic and peri‐domestic rodents from this area (Houéménou et al., 2019).

Our 2 year‐long survey at a finer scale clearly confirms such variations within Cotonou, thus demonstrating spatial inequalities in terms of infectious risks between poor districts of the same city centre. Indeed, the non‐flood‐prone area of Saint‐Jean appeared almost free of rodent‐borne leptospires, while the two insalubrious districts of Agla and Ladji were characterized by significant levels of rodent‐borne *Leptospira*. There, the observed prevalence was moderate (mean values of 11.4% and 14.1% in Agla and Ladji, respectively; 13.2% when the two districts are pooled). However, in all sites that could be sampled during all six sessions (*N* = 12), *Leptospira*‐positive rodents were found at least once, thus confirming widespread circulation of the pathogens within small mammal communities of these two districts. In addition, small mammals in these two neighbourhoods are abundant and omnipresent: overall trapping success reached 18.4% in these two areas while 98.4% (64 out of 65 sites*session) and 85.3% (58 out of 68) of households were found rodent‐infested in Agla and Ladji, respectively (data are not shown). Keeping in mind that a few dozen infected rats may shed thousands of millions of leptospires per day (Costa et al., 2015), our results in Cotonou show that, *in fine*, small mammals represent a potentially massive source of excreted leptospires into the urban environment. Accordingly, pathogenic leptospires were recently detected in Agla and Ladji ponds and wells (Houéménou et al., 2021).

Beyond inter‐neighbourhood differences in prevalence, very local variations were observed between sometimes very close households, too. For instance, comparing only sites for which the six sampling sessions were available (i.e. trapping could be performed and small mammals were captured at each of the six sessions), the 2 year‐long site‐specific prevalence ranged from 5.7% to 25% in Agla, and from 7.3% to 20.6% in Ladji (in addition to one site sampled only three times and reaching a prevalence of 53.3% in the latter locality) (see Table S1). During the same session, site‐specific prevalence could vary from 0% to 100%, although this was usually based on very low sample sizes (Table S1). Nevertheless, these results are in agreement with previous studies suggesting that leptospiral risks strongly depend on local fine‐scale socio‐environmental conditions (Maciel et al., 2008), including in southern Benin (Houéménou et al., 2019).

In some urban settings, *R. norvegicus* was found associated with very high *Leptospira* prevalence (e.g. 80.3% in Salvador, Brazil: Tucunduva de Faria et al., 2008). Accordingly, we found that Norway rats showed the highest species‐specific prevalence (45.2%) in Cotonou. Furthermore, several sites where high small mammal‐borne *Leptospira* prevalence were observed also harboured *R. norvegicus* (e.g. S‐AGL‐8 or S‐LAD‐8; Table S1). However, the species is often associated with shallows and sewers (Dossou et al., 2015; our own observations), thus making it difficult to distinguish between the role of habitat‐ vs. host‐related determinants in favouring the presence of rodent‐borne pathogenic leptospires (Houéménou et al., 2019). Interestingly, other species were also trapped in many of the sites with positive Norway rats, and proved to be *Leptospira*‐positive as well. In addition, Norway rats were not, or very rarely trapped in some other sites where prevalence was quite elevated essentially due to the presence many positive black rats (e.g. S‐AGL‐9 and S‐LAD‐9). Even shrews (that were present in all but two households of Agla and Ladji that could be sampled at least twice) were sometimes found *Leptospira*‐positive in the absence of Norway rats. In the same manner, though limited in numbers, our data show that three out of the four serovars identified were shared by *Rattus rattus* and *R. norvegicus*, and that *L. interrogans* serovar Icterohemorragiae was found in the two rat species as well as in *Mastomys natalensis* and *Crocidura olivieri* (Table 2b). Altogether, these patterns suggest that all small mammals share leptospires widely, and that they may all play a role in *Leptospira* ecology within the Cotonou urban environment. This weakens the hypothesis of partial rodent host‐specificity of *Leptospira* species in South Benin that we recently proposed (Houéménou et al., 2019). Our new results rather point towards the importance of local environmental conditions in determining the possible presence of rodent‐borne leptospires at the household's level (Houéménou et al., 2019). Landscape and health ecology approaches will be useful to further scrutinize these aspects and identify the urban landscape elements that favour *Leptospira* circulation within small mammal assemblages.

Part of the leptospire life cycle takes place in free or soil‐associated waters where they can infect vertebrate hosts (Haake & Levett, 2015). Thus, it seems reasonable to hypothesize that their prevalence in rodents is associated with rainfall and standing waters. Accordingly, some observations were made at the scale of cities and villages of South Benin where prevalence peaks indeed seem to occur during, or 1 month after moderate (100–200 mm) monthly rainfall (Houéménou et al., 2019). Though based on a limited amount of leptospire‐positive water samples collected in Cotonou, isotopic signatures also suggested that leptospires were essentially detected in pond waters formed at the beginning of the rainy season following low to moderate rainfall events (Houéménou et al., 2021). In the present study, very high prevalence was observed during March 2018, where rainfall was moderate though atypically precocious during this particular year (Table 1 and Figure 2). However, no obvious pattern was observed for the other sessions/seasons (Figure 2). In other words, we are unable to confirm conclusively any association between leptospire circulation and (instantaneous or delayed) monthly rainfall.

An interesting result concerns the specific and genotype diversities that were quite high within limited spaces, that is the same neighbourhoods, and even within the same households. Indeed, four different leptospire species and four different genotypes were identified in Agla and Ladji – more than were previously described at the Cotonou and the whole southern Benin spatial scales (Houéménou et al., 2013, 2019). Two examples of two genotypes coexisting at the same moment in rodents from the same household further illustrate the high genetic diversity of small mammal‐borne *Leptospira* in Cotonou. To our knowledge, such a high genetic diversity has been rarely, if ever documented at such a fine scale, especially within urban settings. It also strongly contrasts with what was observed in other settings (see introduction), including the closely located city of Porto‐Novo, Benin, where only *L. interrogans* 16SrDNA sequences were found in 36 qPCR‐positive small mammals (Houéménou et al., 2019).

These marked differences in *Leptospira* specific and genetic diversities among studies may have various origins. Among other non‐exclusive explanations, one may speculate about the plausible importance of the diversity of the host community as well as of potential bioinvasions and introductions of associated leptospire strains. For instance, in Madagascar, a probable spillover of *L. mayottensis* from endemic rodents to the invasive black rat was suggested (Moseley et al., 2018). However, currently available data may not be sufficient to rigorously test these respective hypotheses. Indeed, most molecular investigations of *Leptospira*, such as those conducted in the present study have several caveats in terms of bacterial diversity assessment. First, the characterization of *Leptospira* genotypes from poorly documented regions, such as Africa, often suffers from a lack of baseline microbiological and serological data which could serve as a reference dataset (Mgodé et al., 2015). Here, we combined MST and VNTR profiles, which proved to be quite efficient in obtaining well‐discriminated genotypes. However, their complete taxonomic or serologic identification through comparison to already known profiles is very limited due to the absence of reference data from West Africa.

Second, the coexistence of bacterial strains within the same environment may lead to within‐host coinfections that may be difficult to detect. First, one may imagine that different strains preferentially colonize distinct areas in the host's kidney. To our knowledge, such intra‐host and tissue‐level ecology of *Leptospira* has not been documented, thus precluding addressing this issue further. Second, we found one single *L. borgpetersenii*/*L. interrogans* coinfection in the same *Mastomys natalensis* individual, which echoes the seventeen double and one triple infection recently described in Madagascar (Moseley et al., 2018). However, as in many other studies, we may have under‐detected such coinfection events due to our use of qPCR‐ and sequence‐based approaches, which probably favour the quantitatively dominant strain, and fail to efficiently detect mixed infections. These caveats may represent important biases for studies on the determinants of leptospire species distribution. Yet, we believe that this aspect deserves to be investigated further due to the importance of genetic and immunologic diversity of *Leptospira* in the implementation of any vaccine strategy as well as their probable impact on the aetiology of the infection in both humans and animals. The use of high throughput sequencing may be a promising way to investigate these aspects.

## 5 CONCLUSION

The present study confirms the moderate prevalence of pathogenic *Leptospira* among urban small mammals of South Benin. However, taking into account the strong abundance and density of these reservoir hosts within cities, especially slums, as well as the recurrent flooding episodes that characterized this region, it is expected that inhabitants are at high risk of exposure for leptospirosis (Dobigny et al., 2018). Our longitudinal monitoring documented one of the highest levels of genetic diversity of small mammal‐borne leptospire species and genogroups that has ever been observed at such a fine‐scale, with the co‐occurrence of several genetic *Leptospira* species and strains within the same neighbourhoods and even households. This raises several issues in terms of evolutionary ecology of the pathogen (e.g. host specificity, intra‐host competition, ecological preferences of each *Leptospira* species/strain outside the mammalian host, etc.) as well as disease management (e.g. *Leptospira* species‐ or strain‐specific exposome; design of a locally adapted vaccine). We recommend that raising awareness of the public health authorities, at both the national and local levels, is done urgently, so that studies in humans (including strain typing) are organized, and treatment of leptospirosis is made possible and accessible in Benin.

## CONFLICT OF INTEREST

The authors declare no conflict of interest.

## Supporting information


Figure S1

Figure S2

Figure S3

Table S1

## Data Availability

The data that support the findings of this study are available from the corresponding author upon reasonable request.
