# Supplementary figures and images for: Fine‐scale prevalence and genetic diversity of urban small mammal‐borne pathogenic Leptospira in Africa: A spatiotemporal survey within Cotonou, Benin
Source: Zoonoses Public Health. 2022 May 7;69(6):643–54. doi: 10.1111/zph.12953 (PMC9540415; doi:10.1111/zph.12953)

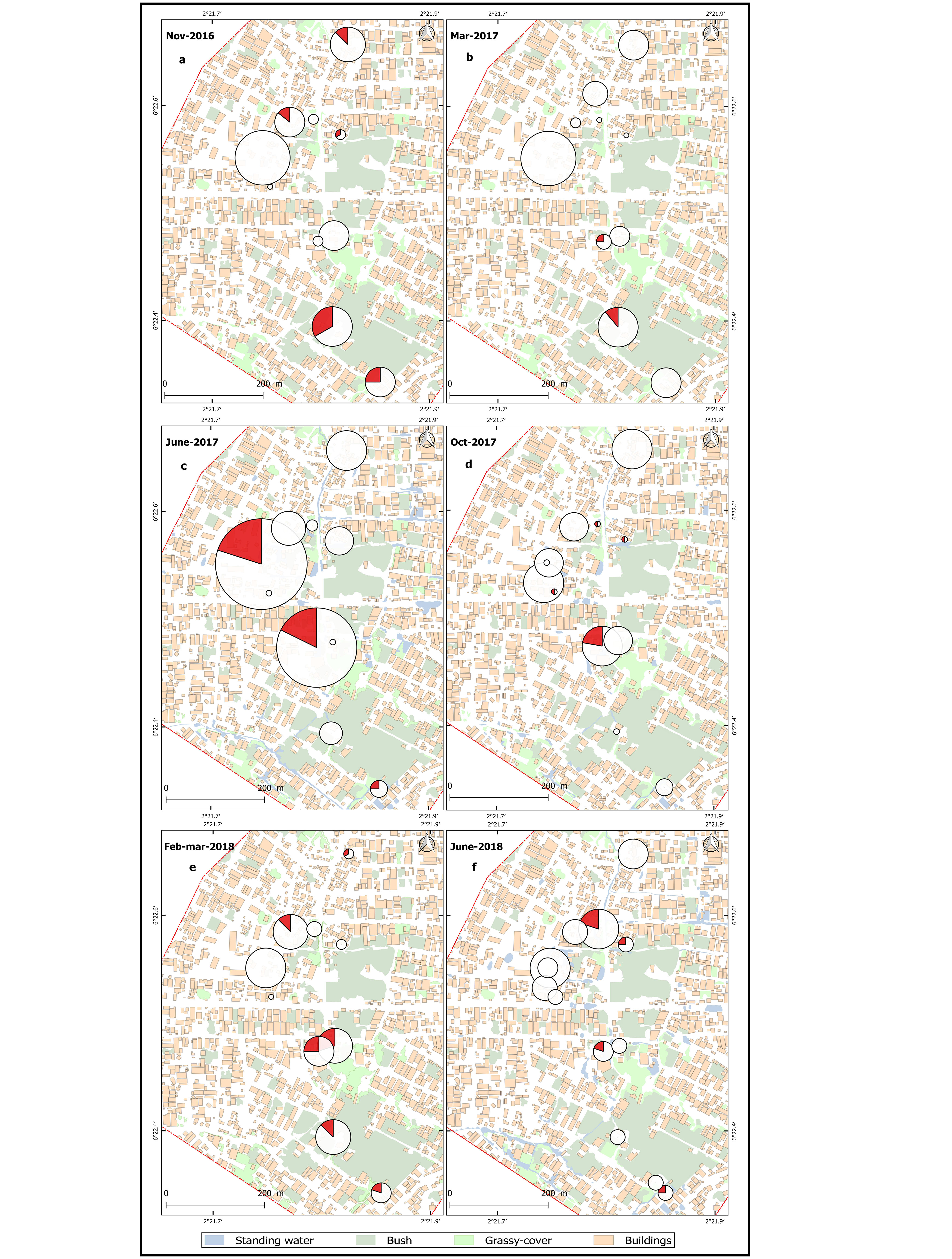

Supplement: Supplementary file 1 — Figure S1 [file ZPH-69-643-s003.jpg]

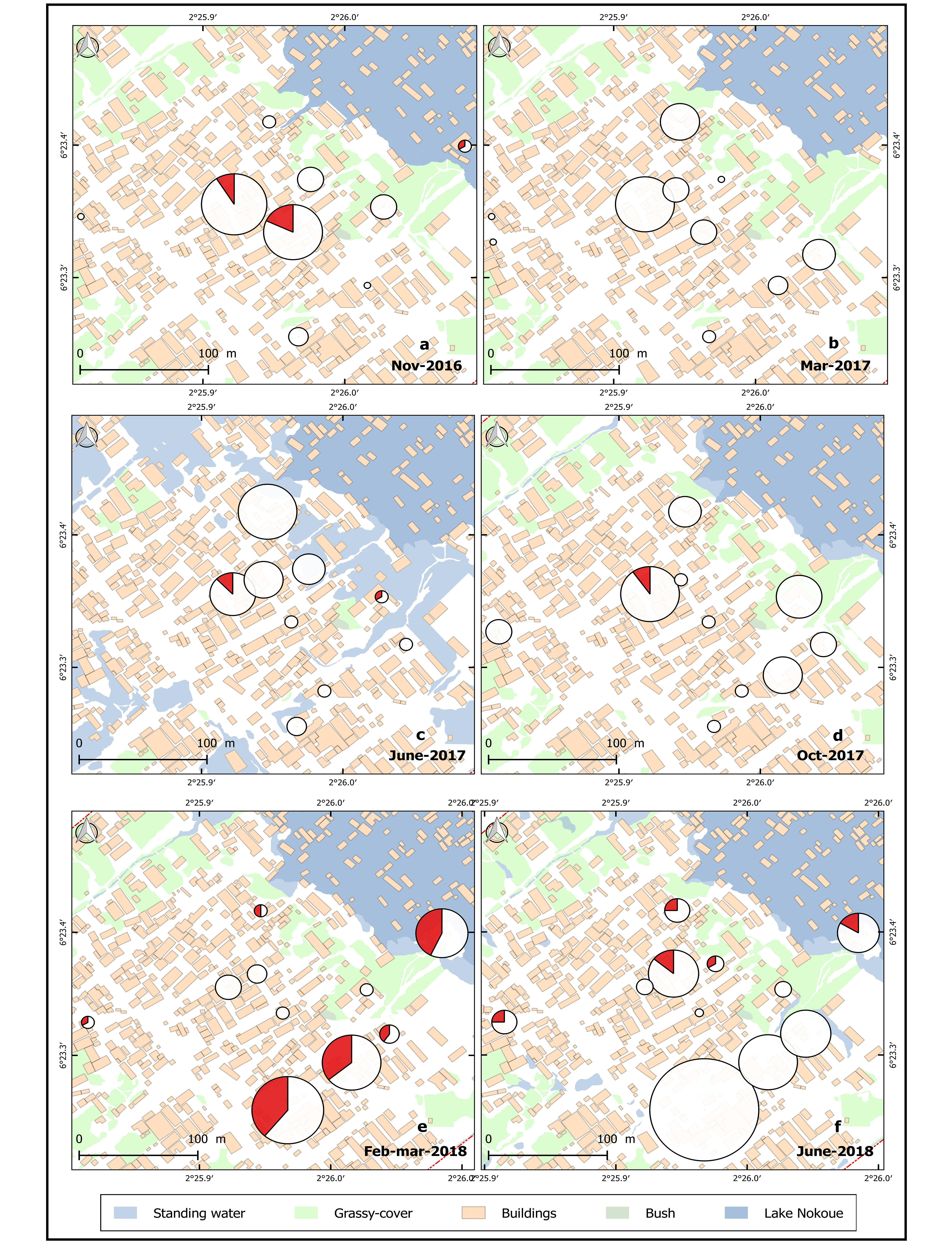

Supplement: Supplementary file 2 — Figure S2 [file ZPH-69-643-s004.jpg]

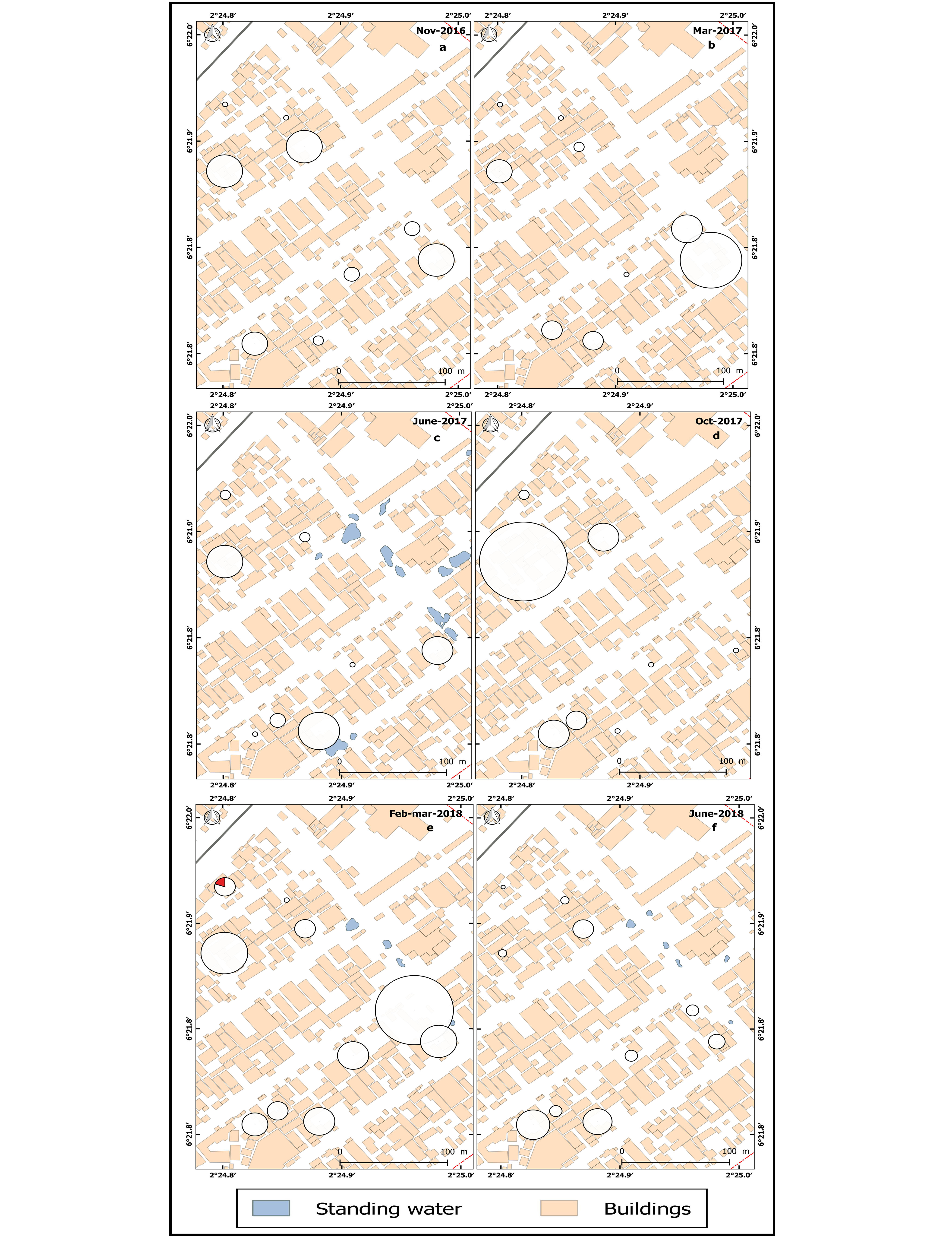

Supplement: Supplementary file 3 — Figure S3 [file ZPH-69-643-s001.jpg]
