# Supplementary material for: Fine‐scale prevalence and genetic diversity of urban small mammal‐borne pathogenic Leptospira in Africa: A spatiotemporal survey within Cotonou, Benin
Source: Zoonoses Public Health. 2022 May 7;69(6):643–54. doi: 10.1111/zph.12953 (PMC9540415; doi:10.1111/zph.12953)
Supplement: Supplementary file 4 — Table S1 [file ZPH-69-643-s002.docx]

**Supplementary Table 1:** Number of captured and qPCR-positive individuals per trapping site, per reservoir species and per session. “Rra”, “Rno”, “Mna”, “Cro”, “Pde” and “Cga” stand for *Rattus rattus*, *R. norvegicus*, *Mastomys natalensis*, *Crocidura olivieri*, *Praomys derooi* and *Cricetomys gambianus*, respectively. “pos” and “prev” correspond to the number of q-PCR positive individuals and the prevalence, respectively. Color codes correspond to prevalence ranges (0-0.25, 0.25-0.5, 0.5-0.75 and 0.75-1).

|  | All sessions | | prev | Nov/Dec 2016 | | | | March 2017 | | | | | June 2017 | | | | | Oct 2017 | | | | Feb/March 2018 | | | | June 2018 | | | |
| --- | --- | --- | --- | --- | --- | --- | --- | --- | --- | --- | --- | --- | --- | --- | --- | --- | --- | --- | --- | --- | --- | --- | --- | --- | --- | --- | --- | --- | --- |
|  | N | pos |  | Rra | Rno | Mna | Cro | Rra | Rno | Mna | Cro | Pde | Rra | Rno | Mna | Cro | Cga | Rra | Rno | Mna | Cro | Rra | Rno | Mna | Cro | Rra | Rno | Mna | Cro |
| S-AGL-1 | 35 | 2 | 0,06 | 1/7 |  |  |  | 0/5 |  |  | 0/1 |  |  |  | 0/4 | 0/3 |  | 0/6 |  |  | 0/1 | 1/2 |  |  |  | 0/2 |  | 0/1 | 0/3 |
| S-AGL-2 | 17 | 3 | 0,18 | 0/2 |  |  |  |  |  |  | 0/1 |  |  |  |  | 0/2 |  |  |  | 1/1 |  |  | 0/1 | 0/1 | 0/1 | 2/3 |  | 0/1 | 0/4 |
| S-AGL-3 | 14 | 3 | 0,21 | 1/2 |  |  |  |  |  | 0/1 |  |  | 0/1 |  | 0/2 | 0/1 | 0/1 |  | 1/1 |  |  | 0/1 |  |  | 0/1 | 1/2 |  |  | 0/1 |
| S-AGL-4 | 7 | 1 | 0,14 |  |  |  | 0/1 | no capture | | | | |  |  |  | 0/1 |  |  |  |  | 1/1 |  |  |  | 0/1 |  |  |  | 0/3 |
| S-AGL-5 | 43 | 4 | 0,09 | 0/1 |  | 0/10 |  |  |  | 0/11 |  |  | 0/1 |  | 4/13 | 0/2 |  |  |  | 0/1 |  | no data | | | | 0/1 |  | 0/2 | 0/1 |
| S-AGL-5' | 21 | 0 | 0,00 | no data | | | | no data | | | | | no data | | | | | 0/2 |  | 0/3 |  | 0/3 |  | 0/5 |  | 0/3 |  | 0/4 | 0/1 |
| S-AGL-5'' | 12 | 0 | 0,00 | no data | | | | no data | | | | | no data | | | | | 0/6 |  |  | 0/1 | no data | | | | 0/2 |  |  | 0/3 |
| S-AGL-6 | 31 | 2 | 0,06 | 1/6 |  |  |  | 0/1 |  |  | 0/1 |  | 0/6 |  |  |  |  | 0/4 |  |  | 0/1 | 1/6 |  |  | 0/1 | 0/2 |  | 0/1 | 0/2 |
| S-AGL-7 | 26 | 3 | 0,12 | 0/6 |  |  |  | 0/3 |  | 0/1 |  |  | 0/1 |  |  |  |  | 0/5 |  |  |  | 3/7 |  |  |  | 0/2 |  |  | 0/1 |
| S-AGL-8 | 36 | 9 | 0,25 | 0/1 | 0/1 |  |  | 1/1 | 0/1 |  | 0/1 |  | 1/7 | 2/7 |  |  |  | 0/4 | 2/3 |  |  |  |  |  | 2/6 |  | 1/2 |  | 0/2 |
| S-AGL-9 | 31 | 6 | 0,19 | 4/7 | 0/1 |  |  | 1/8 |  |  |  |  | 0/4 |  |  |  |  |  | 0/1 |  |  | 0/5 |  |  | 1/2 | 0/1 |  |  | 0/2 |
| S-AGL-10 | 25 | 5 | 0,20 | 1/5 | 1/1 |  |  | 0/6 |  |  |  |  |  | 1/3 |  |  |  |  |  |  | 0/3 |  | 1/1 |  | 0/3 |  | 1/1 |  | 0/2 |
| S-AGL-10' | 3 | 0 | 0,00 | no data | | | | no data | | | | | no data | | | | | no data | | | | no data | | | |  | 0/3 |  |  |
| S-AGL-11 | 5 | 0 | 0,00 | no data | | | | 0/5 |  |  |  |  | no data | | | | | no data | | | | no data | | | | no data | | | |
|  |  |  |  |  |  |  |  |  |  |  |  |  |  |  |  |  |  |  |  |  |  |  |  |  |  |  |  |  |  |
|  | All sessions | | prev | Nov/Dec 2016 | | | | March 2017 | | | | | June 2017 | | | | | Oct 2017 | | | | Feb/March 2018 | | | | June 2018 | | | |
|  | N | pos |  | Rra | Rno | Mna | Cro | Rra | Rno | Mna | Cro | Pde | Rra | Rno | Mna | Cro | Cga | Rra | Rno | Mna | Cro | Rra | Rno | Mna | Cro | Rra | Rno | Mna | Cro |
| S-LAD-1 | 2 | 0 | 0,00 | 0/1 |  |  |  | 0/1 |  |  |  |  | no capture | | | | | no data | | | | no data | | | | no data | | | |
| S-LAD-1' | 10 | 2 | 0,20 | no data | | | | 0/1 |  |  |  |  | no capture | | | | | 0/2 | 0/1 |  | 0/1 | 0/1 | 1/1 |  |  | 1/2 | 0/1 |  |  |
| S-LAD-2 | 20 | 2 | 0,10 | no data | | | | 0/4 |  |  |  | 0/1 | 0/1 |  |  | 0/1 |  |  |  |  | 0/4 |  |  |  | 2/3 | 0/3 |  |  | 0/3 |
| S-LAD-3 | 27 | 3 | 0,11 | 0/2 |  |  |  | 0/6 |  |  |  |  | 0/8 |  |  | 0/1 |  | 0/4 | 0/1 |  |  |  | 2/2 |  |  | 0/1 |  |  | 1/2 |
| S-LAD-4 | 12 | 1 | 0,08 | 0/4 |  |  |  | 0/1 |  |  |  |  | 0/5 |  |  |  |  | no data | | | | no data | | | | 1/2 |  |  |  |
| S-LAD-4' | 21 | 1 | 0,05 | no data | | | | 0/4 |  |  |  |  | 0/5 |  |  | 0/1 |  | 0/1 |  | 0/1 |  | 0/3 |  |  |  | 0/5 |  |  | 1/1 |
| S-LAD-5 | 20 | 2 | 0,10 | 1/6 | 1/1 |  | 0/2 | 0/3 | 0/1 |  |  |  |  |  |  | 0/2 |  | 0/1 |  |  | 0/1 |  |  |  | 0/2 |  |  |  | 0/1 |
| S-LAD-6 | 26 | 5 | 0,19 | 0/1 |  |  |  | 0/3 |  |  |  |  | no capture | | | | | 0/5 |  |  | 0/1 | 5/9 |  |  |  | 0/3 |  |  | 0/4 |
| S-LAD-7 | 17 | 1 | 0,06 | 0/4 |  |  |  | no capture | | | | | 0/1 |  | 1/1 |  |  | 0/6 |  |  | 0/1 | 0/1 |  |  | 0/1 | 0/1 |  |  | 0/1 |
| S-LAD-8 | 15 | 8 | 0,53 | 1/2 |  |  |  | no capture | | | | | no capture | | | | | no data | | | |  | 4/4 |  | 2/4 |  | 1/1 |  | 0/4 |
| S-LAD-9 | 34 | 7 | 0,21 | 0/1 |  | 0/1 | 0/1 | 0/2 |  |  |  |  | 0/1 |  |  | 0/2 |  | 0/2 |  |  |  | 7/11 |  |  |  | 0/10 |  |  | 0/3 |
| S-LAD-10 | 41 | 3 | 0,07 | 1/10 |  |  |  | 0/9 |  |  |  |  | 1/6 |  |  | 0/1 |  | 1/8 |  |  | 0/1 | 0/3 |  |  | 0/1 | 0/1 |  |  | 0/1 |
| S-LAD-11 | 4 | 0 | 0,00 | no data | | | | no data | | | | |  | 0/2 |  |  |  |  |  | 0/2 |  | no data | | | | no capture | | | |
|  |  |  |  | 0-0.25 | | 0.25-0.5 | | 0.5-0.75 | | 0.75-1 | |  |  |  |  |  |  |  |  |  |  |  |  |  |  |  |  |  |  |
